# Comparison of the vitamin D level between children with and without cow's milk protein allergy: a systematic review with meta-analysis

**DOI:** 10.3389/fped.2025.1649825

**Published:** 2025-09-11

**Authors:** Xueming Zheng, Yi Jin, Jianhong Luo, Fan Jiang, Limin Zhang, Xiaoxue Wang, Hui Zhang, Shaofei Ma, Yejuan Li

**Affiliations:** ^1^ Child Health Section, Xingtai People’s Hospital, Xingtai, China; ^2^ Pediatric Respiratory Department, Hunan Provincial People’s Hospital, Changsha, China; ^3^ School of Environment and Health, Yanching Institute of Technology, Langfang, China; ^4^ Department of Nephrology, The Second Hospital of Hebei Medical University, Shijiazhuang, China

**Keywords:** cow's milk protein allergy, vitamin D, children, immune function, meta-analysis

## Abstract

**Background:**

Vitamin D plays an important role in modulating immune responses, which may be associated with cow's milk protein allergy (CMPA). This meta-analysis aimed to comprehensively compare the vitamin D level between children with CMPA and healthy controls.

**Methods:**

Studies comparing the vitamin D level between children with CMPA and healthy controls were comprehensively searched in PubMed, Web of Science, Embase, Wan Fang, CNKI, and VIP until April 2025.

**Results:**

A total of 12 studies, involving 605 children with confirmed CMPA (CMPA group) and 558 healthy controls (control group) were included. The vitamin D level was lower in the CMPA group than in the control group [standardized mean difference (SMD) (95% confidence interval): −1.229 (−2.117, −0.340), *P* = 0.007]. Regarding subgroup analysis, the vitamin D level was lower in the CMPA group than in the control group in studies using mass spectrometry or automated immunoassay (*P* = 0.042), and was of no difference in those using enzyme-linked immunosorbent assay (*P* = 0.200) or chemiluminescence (*P* = 0.097). Moreover, the vitamin D level was lower in the CMPA group than in the control group in studies conducted in Asia (*P* < 0.001) or South America (*P* = 0.041), but not in studies conducted in Europe (*P* = 0.500). All included studies were high-quality. No publication bias was found. Sensitivity analyses indicated high robustness of the results.

**Conclusion:**

Vitamin D is decreased in children with CMPA, indicating its potential association with CMPA. These findings may enhance the understanding of CMPA and guide the prevention or management of CMPA in children.

## 1 Introduction

Cow's milk protein allergy (CMPA) refers to an immune-mediated reaction to cow's milk proteins (1, 2). CMPA is mainly classified as immunoglobulin E (IgE)-mediated and non-IgE-mediated subtypes (3). IgE-mediated CMPA manifests with immediate symptoms such as acute urticaria, angioedema, and vomiting, while non-IgE-mediated CMPA presents with delayed symptoms such as chronic diarrhea and bloody stools (4, 5). According to the recent 2024 European Society of Paediatric Gastroenterology, Hepatology and Nutrition guideline, the prevalence of CMPA in infants and children is <1% around the world, and it varies across different regions (such as <0.3% in Lithuania, Germany and Greece and 1% in the Netherlands and United Kingdom) (6). CMPA not only negatively influences the quality of life of children but also poses a psychosocial and economic burden to their families (7, 8). Exploring potential biomarkers may further contribute to the prevention or management of CMPA.

Vitamin D is an essential micronutrient for the human body, and plays a vital role in the regulation of immune responses (9, 10). Currently, vitamin D deficiency has been considered to be associated with the occurrence and progression of food allergies, including CMPA (11, 12). The causality between vitamin D deficiency and CMPA could be bidirectional. On one hand, vitamin D deficiency may prevent vitamin D from binding to the vitamin D receptor on immune cells, which promotes pro-inflammatory pathways and causing immune dysregulation, thus leading to CMPA (13). On the other hand, CMPA may result in vitamin D deficiency because it causes malabsorption and induces the development of nutritional disorders (14). However, previous studies have shown inconsistent findings on the association between vitamin D and CMPA (15–26). Most studies found that the vitamin D level of children with CMPA was lower than that of healthy controls (16, 17, 21–26). While some studies reported that there was no difference in the vitamin D level between children with CMPA and healthy controls (15, 18–20). Thus, it is necessary to conduct a comprehensive analysis to further assess the association of vitamin D with CMPA.

Therefore, the present meta-analysis aimed to systematically compare the vitamin D level between children with CMPA and healthy controls.

## 2 Material and methods

### 2.1 Search approach

This meta-analysis was conducted per the Preferred Reporting Items for Systematic Reviews and Meta-Analyses (PRISMA) guidelines. To ensure comprehensive coverage of relevant literature, we searched several major biomedical databases, including international databases (PubMed, Web of Science, and Embase) and well-known Chinese academic databases (Wan Fang, CNKI, and VIP). The search timeframe spanned from database inception through April 2025 to capture the most contemporary evidence. The search strategy combined key terms as follows: “vitamin D”, “25-hydroxyvitamin D”, “25(OH)D”, “cow's milk protein allergy”, “cow's milk allergy”, “milk protein allergy”, “CMPA”, “child”, “kid”, “infant”, and “baby”. Additional relevant studies were identified by manually screening the reference lists of the included articles.

### 2.2 Screening criteria

The inclusion criteria were as follows: (1) studies reported children with confirmed CMPA; (2) studies reported comparisons of the vitamin D level [assessed by 25(OH)D concentration] between CMPA and healthy controls (Control); (3) studies reported sufficient data on the vitamin D level for effect size estimation; and (4) studies published in English or Chinese. Studies were excluded if they (1) included subjects with multiple food allergies without isolated CMPA data; (2) lacked a control group; or (3) were reviews, meta-analyses, case reports, animal studies, or experiments.

### 2.3 Information extraction and quality appraisal

The first author, publication year, region (continent), study design, participant characteristics, sample size, and the detective method of vitamin D were independently extracted by two reviewers. Discrepancies were adjudicated through iterative discussion until consensus was achieved. Study quality was assessed using the Newcastle-Ottawa Scale (NOS) (27). Studies scoring ≥6 on NOS were considered high quality.

### 2.4 Statistical analysis

The comparative analysis of the vitamin D level between CMPA and Control was performed via the pooled standardized mean difference (SMD) with a 95% confidence interval (CI). Between-study heterogeneity was examined through the Cochran *Q*-test (*P* < 0.10 indicating significant heterogeneity) and quantified by the I² statistic (I² more than 50%). The random-effect model was applied for the pooled vitamin D level. To explore potential effect modifiers, pre-specified subgroup analyses were implemented according to continent and detective method of vitamin D. Methodological robustness was verified through leave-one-out sensitivity analyses, systematically excluding individual studies to evaluate result stability. Publication bias assessment incorporated both graphical inspection of a funnel plot and statistical evaluation via Egger's and Begg's tests (statistical significance: *P* < 0.05). All the statistical analyses were performed via R software (version 4.4.2).

## 3 Results

### 3.1 Study screening process

**Figure 1:**
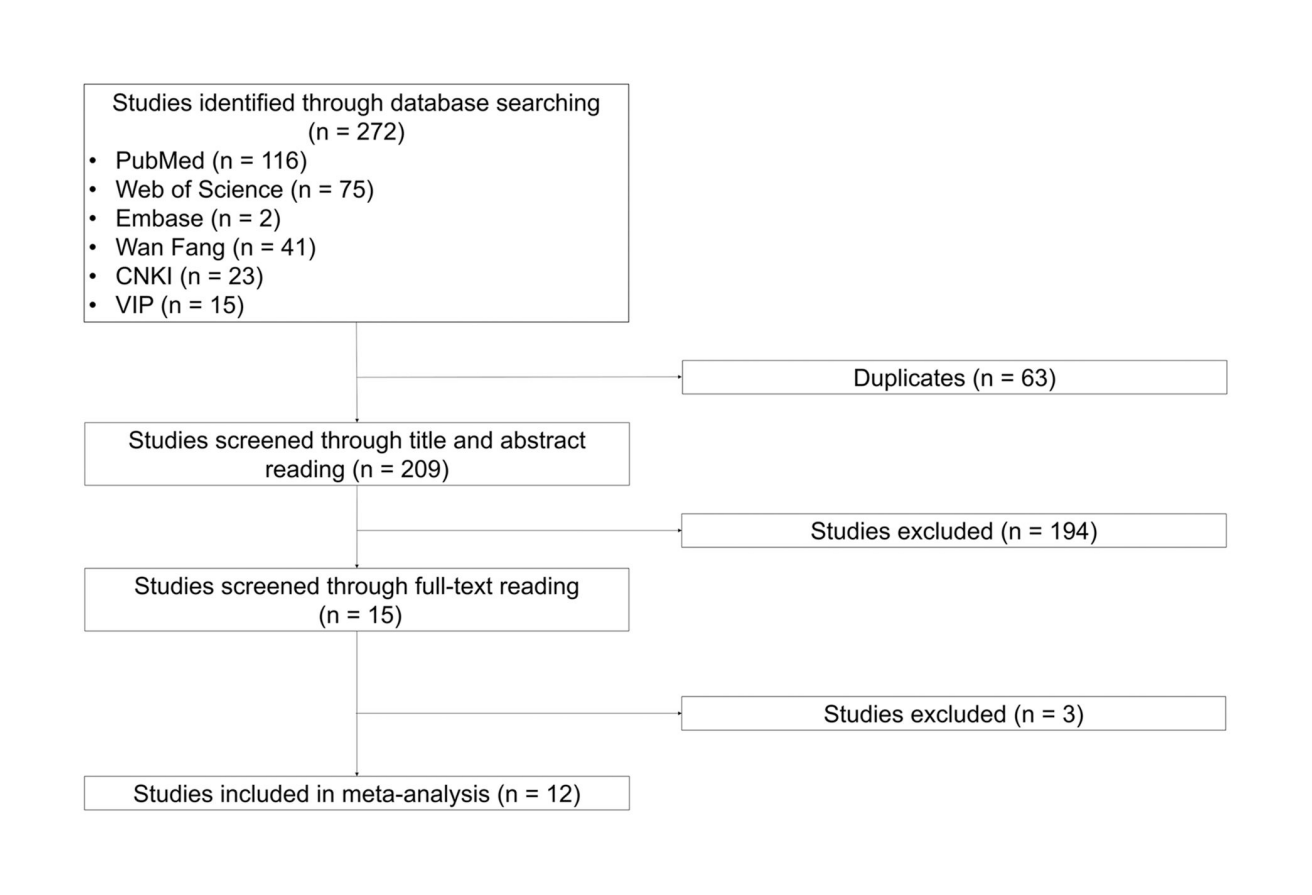
Flow chart of study screening.

A total of 272 studies were identified through database searching, of which 116 were from PubMed, 75 were from Web of Science, 2 were from Embase, 41 were from Wan Fang, 23 were from CNKI, and 15 were from VIP. Then, 63 duplicated studies were excluded. After the title and abstract were read, 194 studies were removed. Subsequently, 3 studies were excluded through full-text reading. Eventually, the remaining 12 studies were included in this meta-analysis (15–26) (Figure 1).

### 3.2 Study features

The 12 studies were published between 2014 and 2022, and included 605 children with confirmed CMPA (CMPA group) and 558 healthy controls (control group). In most studies, the median age of participants ranged from 2.2 to 24 months, except for one study, which included patients ranging from 3 to 6 years (median age: 4.7 years). Among the included studies, 5 studies were conducted in Europe, 6 studies were conducted in Asia, and 1 study was conducted in South America. With respect to study design, 3 studies were cross-sectional, and the others were case-control studies. In terms of the detective method of vitamin D, 5 studies used enzyme-linked immunosorbent assay (ELISA), 5 studies applied chemiluminescence, and 2 studies used other methods. More specific information is presented in Table 1. Moreover, the diagnosis criteria of CMPA and the elimination diet information of included studies is listed in Supplementary Table S1.

**Table 1:** Characteristics of studies.

Study	Continent	Study design	Sample size		Age (months)		Sex (boy/girl)		Detective method of Vitamin D
			CMPA group	Control group	CMPA group	Control group	CMPA group	Control group	
Ambroszkiewicz et al. (15)	Europe	Case-control	50	40	Mean: 4.7^a^	Mean: 4.7^a^	33/17	23/17	ELISA
Perezabad et al. (16)	Europe	Case-control	15	13	Mean: 6.4	Mean: 6.2	9/6	7/6	Chemiluminescence
Silva et al. (17)	South America	Cross-sectional	59	61	Median: 7.0	Median: 9.0	25/34	30/31	Chemiluminescence
Ercan et al. (18)	Europe	Case-control	56	55	Mean: 7.3	Mean: 7.8	32/24	36/19	Chemiluminescence
Yang et al. (19)	Asia	Case-control	30	30	Mean: 2.2	Mean: 2.6	14/16	13/17	Chemiluminescence
Dogan and Sevinc (20)	Europe	Case-control	62	58	Median: 4.0	Median: 4.5	39/21	32/26	ELISA
Pandiaraja and Maris (21)	Europe	Cross-sectional	52	26	Median:16.0	Median: 11.0	25/27	14/12	Mass spectrometry or automated immunoassay
Zhou et al. (22)	Asia	Case-control	50	50	39 cases <7.0				
11 cases ≥7.0	40 cases <7.0								
10 cases ≥7.0	30/20	29/21	ELISA						
Che et al. (23)	Asia	Cross-sectional	41	50	Below 24.0	Below 24.0	24/17	24/26	ELISA
Li and Wu (24)	Asia	Case-control	32	17	Mean: 3.6	Mean: 3.5	13/19	5/12	ELISA
Li et al. (25)	Asia	Case-control	102	102	4.0∼8.0	NA	NA	NA	Mass spectrometry
Peng and Liu (26)	Asia	Case-control	56	56	Mean: 6.2	Mean: 6.4	24/32	26/30	Chemiluminescence

CMPA, cow's milk protein allergy; ELISA, enzyme-linked immunosorbent assay; NA, not available.

^a^ The unit was “years”.

### 3.3 Study quality

The quality assessment of the included studies was conducted via the NOS. There were 3 studies with a total score of 8 and 9 studies with a total score of 9. This indicated that all the included studies were of high quality (Table 2).

**Table 2:** Quality assessment.

Study	Selection	Comparability	Outcome	Total
Ambroszkiewicz et al. (15)	4	2	3	9
Perezabad et al. (16)	3	2	3	8
Silva et al. (17)	3	2	3	8
Ercan et al. (18)	4	2	3	9
Yang et al. (19)	3	2	3	8
Dogan and Sevinc (20)	4	2	3	9
Pandiaraja and Maris (21)	4	2	3	9
Zhou et al. (22)	4	2	3	9
Che et al. (23)	4	2	3	9
Li and Wu (24)	4	2	3	9
Li et al. (25)	4	2	3	9
Peng and Liu (26)	4	2	3	9

### 3.4 Comparison of the vitamin D level between groups

**Figure 2:**
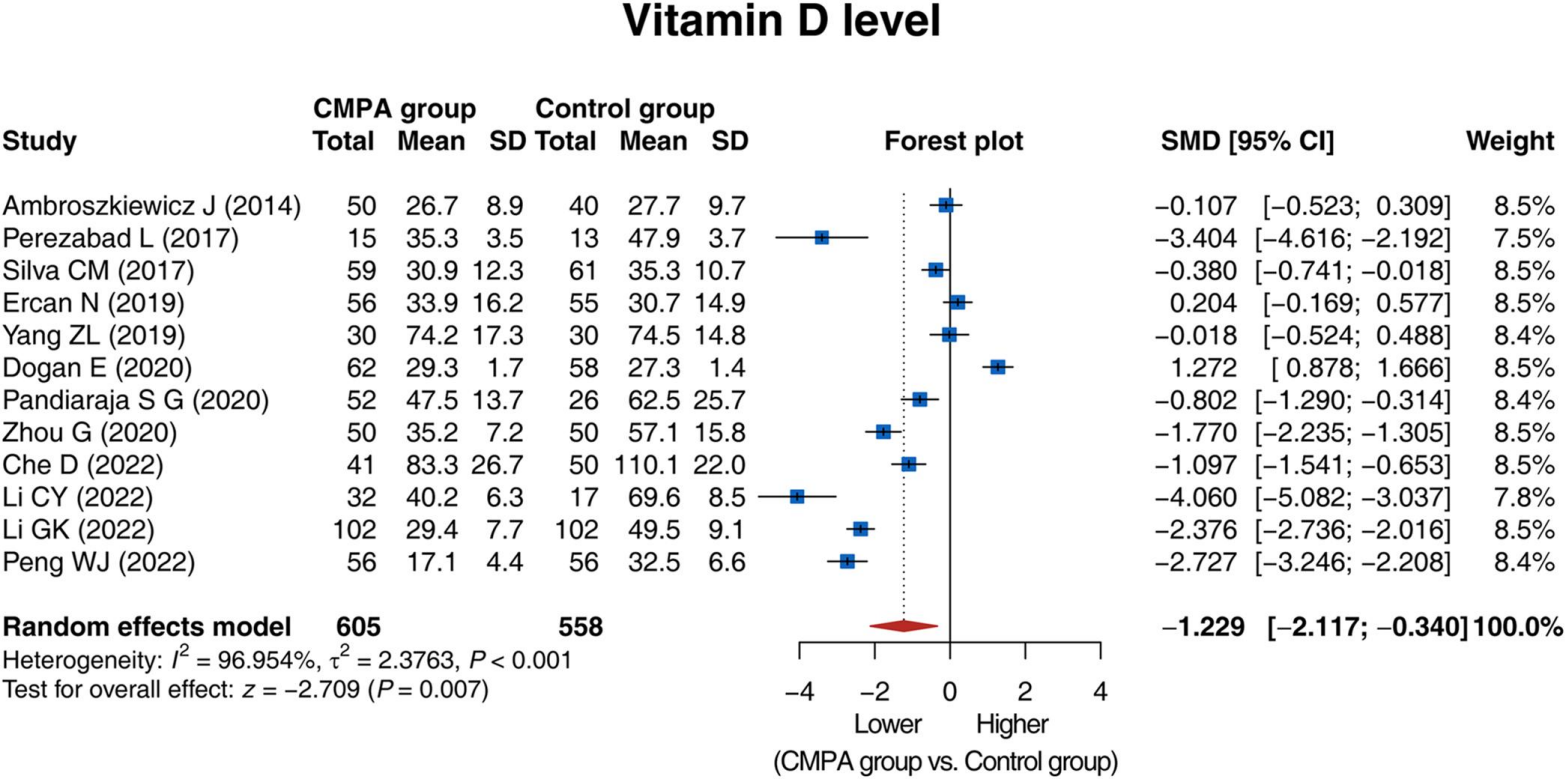
Forest plot of the comparison of the vitamin D level between groups.

The 12 studies compared the vitamin D level between the CMPA group and the control group, and there was heterogeneity (I^2^ = 96.954%, *P* < 0.001). The random effects model revealed that the vitamin D level was lower in the CMPA group than in the control group [SMD (95% CI): −1.229 (−2.117, −0.340), *P* = 0.007] (Figure 2).

### 3.5 Subgroup analysis for comparison of the vitamin D level between groups

**Figure 3:**
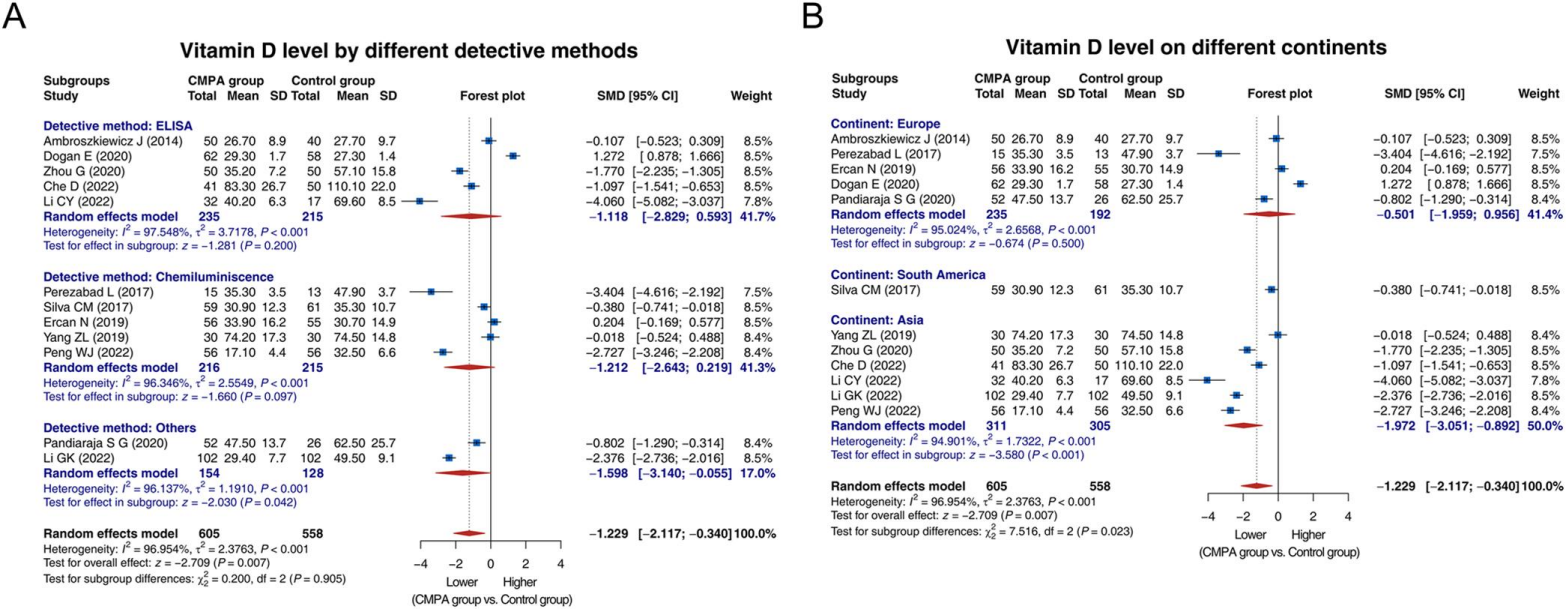
Forest plots of subgroup analysis for comparison of the vitamin D level between groups. Subgroup analysis for comparison of the vitamin D level between groups based on different detective methods **(A)** and different continents **(B)**.

Subgroup analysis was conducted based on different detective methods. Five studies used ELISA, and heterogeneity was observed among these studies (I^2^ = 97.548%, *P* < 0.001). The random effects model showed that the vitamin D level tended to be lower in the CMPA group than in the control group, while there was no statistical significance [SMD (95% CI): −1.118 (−2.829, 0.593), *P* = 0.200]. Five studies detected vitamin D level by chemiluminescence, and there was heterogeneity among these studies (I^2^ = 96.346%, *P* < 0.001). The random effects model disclosed that the vitamin D level tended to be lower in the CMPA group than in the control group, while not reaching statistical significance [SMD (95% CI): −1.212 (−2.643, 0.219), *P* = 0.097]. Two studies used other methods to detect vitamin D, and there was heterogeneity (I^2^ = 96.137%, *P* < 0.001). The random effects model suggested that the vitamin D level was lower in the CMPA group than in the control group [SMD (95% CI): −1.598 (−3.140, −0.055), *P* = 0.042] (Figure 3A).

Regarding subgroup analysis based on different continents, 5 studies were conducted in Europe, and heterogeneity existed among these studies (I^2^ = 95.024%, *P* < 0.001). The random effects model showed no difference in the vitamin D level between groups [SMD (95% CI): −0.501 (−1.959, 0.956), *P* = 0.500]. One study conducted in South America suggested that the vitamin D level was lower in the CMPA group than in the control group [SMD (95% CI): −0.380 (−0.741, −0.018), *P* = 0.041]. A total of 6 studies were conducted in Asia, and heterogeneity was found among these studies (I^2^ = 94.901%, *P* < 0.001). The random effects model suggested that the vitamin D level was lower in the CMPA group than in the control group [SMD (95% CI): −1.972 (−3.051, −0.892), *P* < 0.001] (Figure 3B).

### 3.6 Publication bias and sensitivity analysis

**Figure 4:**
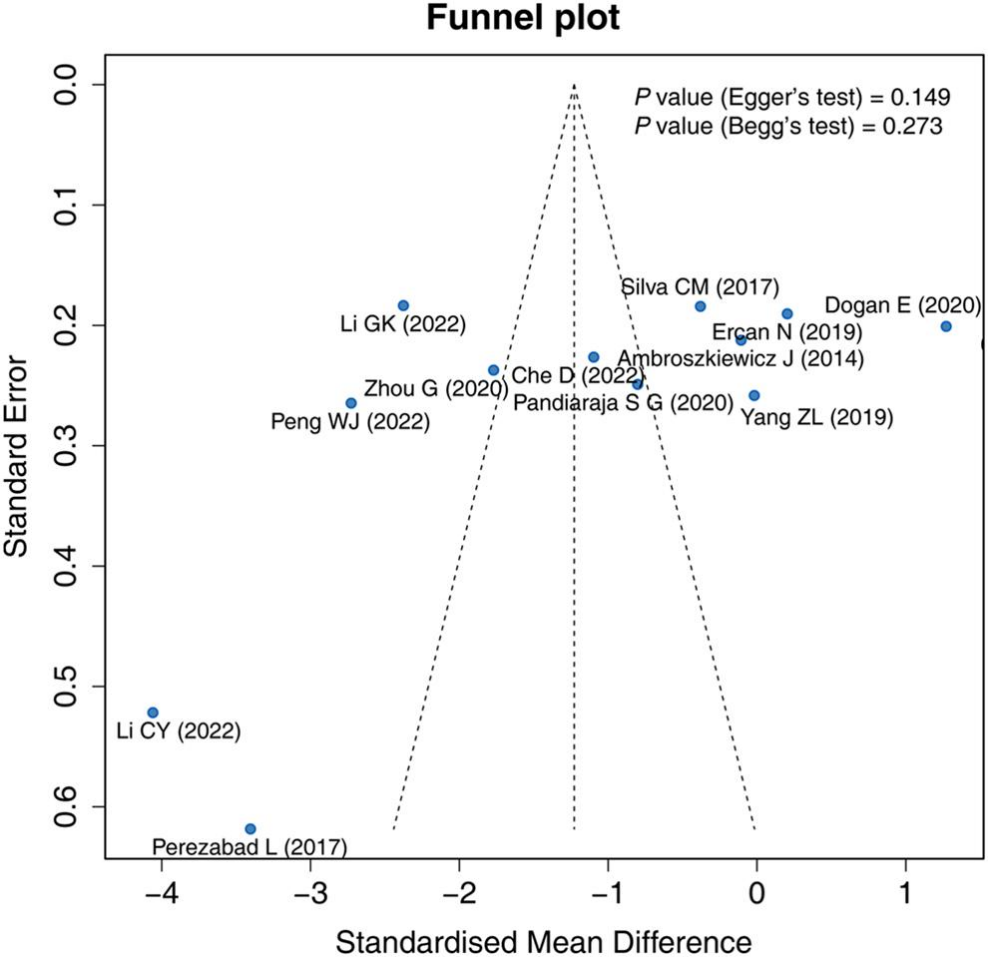
Funnel plot.

The funnel plot, Egger's test, and Begg's test were applied to evaluate publication bias. The funnel plot exhibited that the included studies were roughly symmetrically distributed, indicating that there was no significant publication bias among the studies. Egger's test (*P* = 0.149) and Begg's test (*P* = 0.273) showed that there was no publication bias (Figure 4).

**Figure 5:**
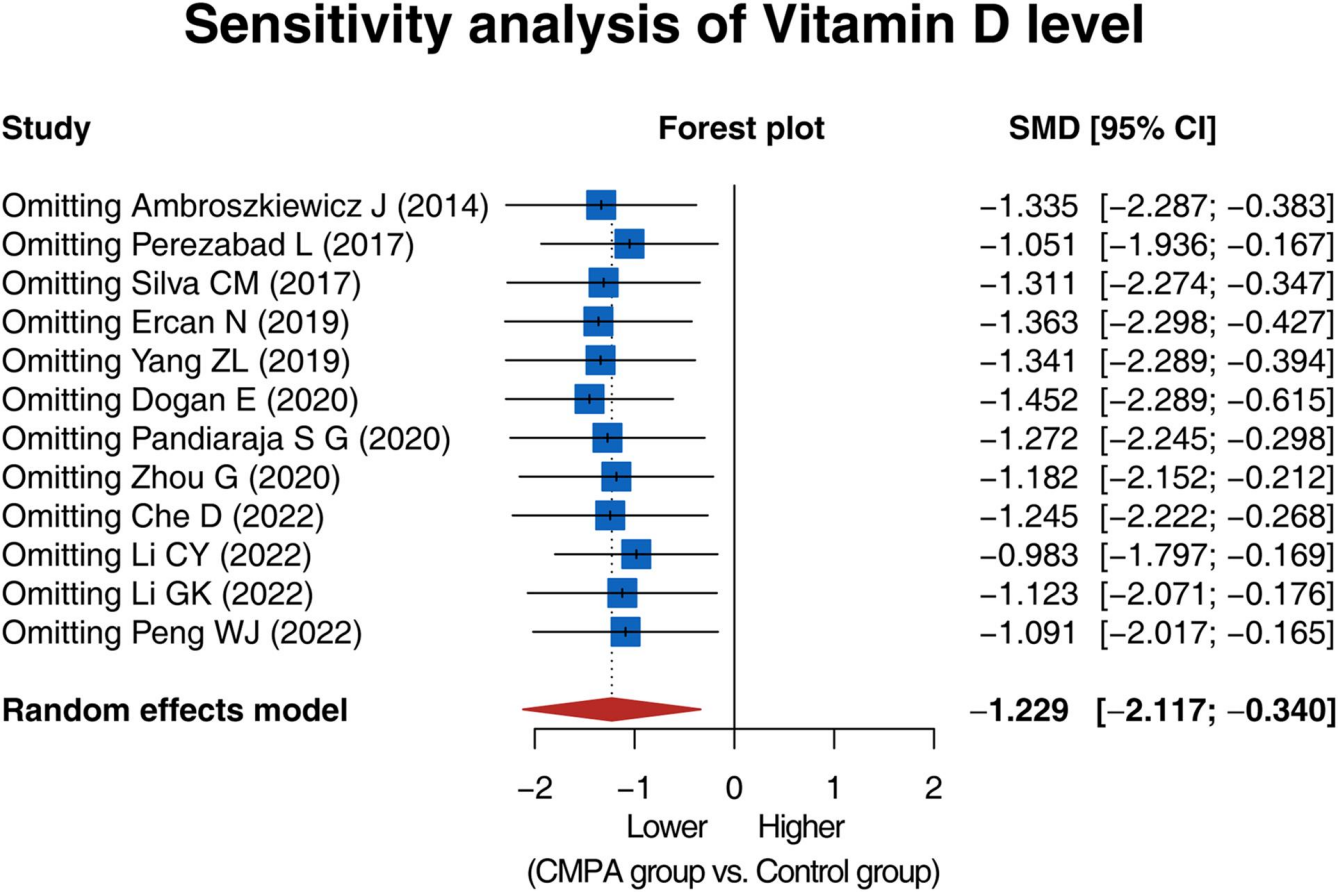
Forest plot of sensitivity analysis.

Sensitivity analysis showed that the result of the comparison of the vitamin D level between groups would not be influenced by removing any single study, indicating a high robustness of the result (Figure 5).

## 4 Discussion

The pathogenesis of CMPA involves abnormal immune function in the body (28). Vitamin D can maintain the stability of the immune system by binding to the vitamin D receptor expressed on various immune cells (10, 29, 30). In detail, vitamin D enhances innate immunity by increasing the chemotaxis and phagocytosis capabilities of monocytes and macrophages, as well as inducing the production of antimicrobial peptides (29, 31). In the adaptive immune system, vitamin D stimulates the differentiation of T helper cell 2 (Th2) and regulatory T cells, while inhibiting the differentiation of Th1 and Th17 cells (29, 32). Moreover, vitamin D also regulates B-cell activity and IgE production (29). Based on the above contents, it is hypothesized that there is a potential association of vitamin D with CMPA. However, the conclusions of previous studies were controversial (15–26). This meta-analysis comprehensively reviewed data from previous studies and revealed that the vitamin D level was lower in children with CMPA than in healthy controls. The result indicated that vitamin D deficiency might be closely related to CMPA.

Notably, there was high heterogeneity among the studies included in this meta-analysis. To further assess the sources of heterogeneity for the included studies, this meta-analysis conducted subgroup analyses based on different detective methods and different continents. Regarding the detective methods, mass spectrometry is the gold standard with high sensitivity and specificity, but it has disadvantages such as a (semi)manual sample preparation, the need for expensive instrumentation and experienced staff, and is not yet suitable for high throughput; automated immunoassay shows high variability in clinical detection; ELISA and chemiluminescence are operationally easy with low cost, while their performance is inferior to mass spectrometry (33, 34). The results showed that the vitamin D level was lower in children with CMPA than in healthy controls in studies that detected the vitamin D level by mass spectrometry or automated immunoassay. In studies measuring the vitamin D level by ELISA or chemiluminescence, the vitamin D level tended to be lower in children with CMPA than in healthy controls, while there was no statistical significance. This might be due to the inconsistent results from individual studies influencing the results of the subgroup (15, 18–20). Meanwhile, the test for subgroup difference showed that there was no statistical significance in the result of the comparison of the vitamin D level between children with CMPA and healthy controls among subgroups. Thus, the detective methods might not be the source of heterogeneity. Moreover, the vitamin D level showed a discrepancy between children with CMPA and healthy controls in studies conducted in Asia or South America, while no difference was observed in studies conducted in Europe. This finding suggested that different continents might be one of the sources of heterogeneity. In detail, the association between vitamin D and CMPA might be regionally dependent, and monitoring vitamin D might be more important for children with CMPA in Asia and South America. However, there was still high heterogeneity in each subgroup. This result indicated that there might also be other sources of heterogeneity among these studies, such as baseline characteristics.

Based on the above findings, two hypotheses were proposed: (1) Vitamin D deficiency increased the risk of CMPA. (2) CMPA resulted in vitamin D deficiency in children. The result of our meta-analysis was unable to determine whether vitamin D was the cause or the consequence of CMPA, and further studies were required. However, regardless of which hypothesis is correct, routine vitamin D screening and supplementation are important for children. The European Academy of Paediatrics recommends screening vitamin D status in high-risk children, and the World Allergy Organization recommends vitamin D supplementation for children with CMPA (35, 36). In the context of CMPA, vitamin D promotes the development of regulatory T-cells, which maintains the balance of immune response and prevents excessive inflammatory reactions (37, 38). Notably, vitamin D cannot be synthesized directly by the body, and it is obtained through two main sources: the diet and exposure to ultraviolet B rays (22, 39). Therefore, vitamin D supplementation and appropriate sunlight exposure are recommended for children to increase the vitamin D level, which could be considered a potential management strategy for CMPA (40, 41).

The quality assessment via the NOS method disclosed that all included studies in this meta-analysis were of high quality. Moreover, no publication bias was observed. Sensitivity analysis showed a high robustness of the result. Nevertheless, several limitations should be noted in this meta-analysis: (1) There was high heterogeneity among studies (most I² > 90.0%), which limited the strength of the findings. Although subgroup analysis and sensitivity analysis were performed, the sources of heterogeneity were still not fully explained. (2) This meta-analysis included only studies published in English or Chinese. Future studies should consider including studies published in other languages for further verification. (3) This meta-analysis only compared the vitamin D level between children with CMPA and healthy controls, which is not enough to provide a deep understanding of the causal relationship between vitamin D and CMPA. Further study about the deep mechanism of vitamin D in regulating the pathogenesis of CMPA should be carried out (4) There might be some confounding factors influencing the vitamin D level, such as seasonal variation, the use of vitamin D supplementation, dietary intake, and sun exposure (42, 43). However, the corresponding information in the included studies was not detailed enough for pooled analysis. Future studies should consider these confounding factors for further verification.

## 5 Conclusion

In conclusion, the vitamin D level is lower in children with CMPA. The findings of this meta-analysis reveal a potential association of vitamin D with CMPA. However, high heterogeneity among included studies may limit the strength of our findings. Overall, timely vitamin D screening and supplementation are recommended for children, which may be helpful in the prevention or management of CMPA.

## Data Availability

The original contributions presented in the study are included in the article/Supplementary Material, further inquiries can be directed to the corresponding author.
