# Supplementary material for: Comparison of the vitamin D level between children with and without cow's milk protein allergy: a systematic review with meta-analysis
Source: Front Pediatr. 2025 Sep 11;13:1649825. doi: 10.3389/fped.2025.1649825 (PMC12460241; doi:10.3389/fped.2025.1649825)
Supplement: Supplementary file 1 [file Table1.docx]

**Supplementary Table 1.** Supplementary characteristics of studies.

| Study | CMPA diagnosis | Elimination diet | |
| --- | --- | --- | --- |
|  |  | CMPA group | Control group |
| Ambroszkiewicz J (2014)(15) | The presence of clinical symptoms of an allergy, elevated serum-specific immunoglobulin E, positive results of skin-prick test, and positive results of open oral milk challenge test | A dairy-free diet for at least a year, and remained under medical and nutritional care | Not taking any bone sparing drugs |
| Perezabad L (2017) (16) | Oral challenge test (unless contraindicated by severe clinical profile), and considering the total and specific IgE values, physical examination, and family history | NR | NR |
| Silva CM (2017) (17) | Considering the clinical history, physical examination, and previous history, and confirmed by the open oral challenge test | NR | NR |
| Ercan N (2019) (18) | Oral food challenge test | Not received | Not received |
| Yang ZL (2019) (19) | Oral food challenge test | Nutritional management using amino acid-based or extensively hydrolyzed formulas | Not received |
| Dogan E (2020) (20) | Considering the clinical history, physical examination, and previous history, and confirmed by the open oral challenge test, skin prick tests, and specific IgE measurements | A therapeutic CMPA elimination diet | Not received |
| Pandiaraja S G (2020) (21) | Based on a suggestive clinical history and confirmed by skin prick tests, and specific IgE measurements | Received CMPA elimination diet | Not received |
| Zhou G (2020) (22) | Evidence-based Recommendations for Diagnosis and Management of Cow's Milk Protein Allergy in Chinese Infants | NR | NR |
| Che D (2022) (23) | Oral food challenge test | Not received | Not received |
| Li CY (2022) (24) | Evidence-based Recommendations for Diagnosis and Management of Cow's Milk Protein Allergy in Chinese Infants | NR | NR |
| Li GK (2022) (25) | Evidence-based Recommendations for Diagnosis and Management of Cow's Milk Protein Allergy in Chinese Infants | NR | NR |
| Peng WJ (2022) (26) | Evidence-based Recommendations for Diagnosis and Management of Cow's Milk Protein Allergy in Chinese Infants | NR | NR |

CMPA, cow’s milk protein allergy; NR, not reported.
