# Supplementary figures and images for: Comparison of the vitamin D level between children with and without cow's milk protein allergy: a systematic review with meta-analysis
Source: Front Pediatr. 2025 Sep 11;13:1649825. doi: 10.3389/fped.2025.1649825 (PMC12460241; doi:10.3389/fped.2025.1649825)

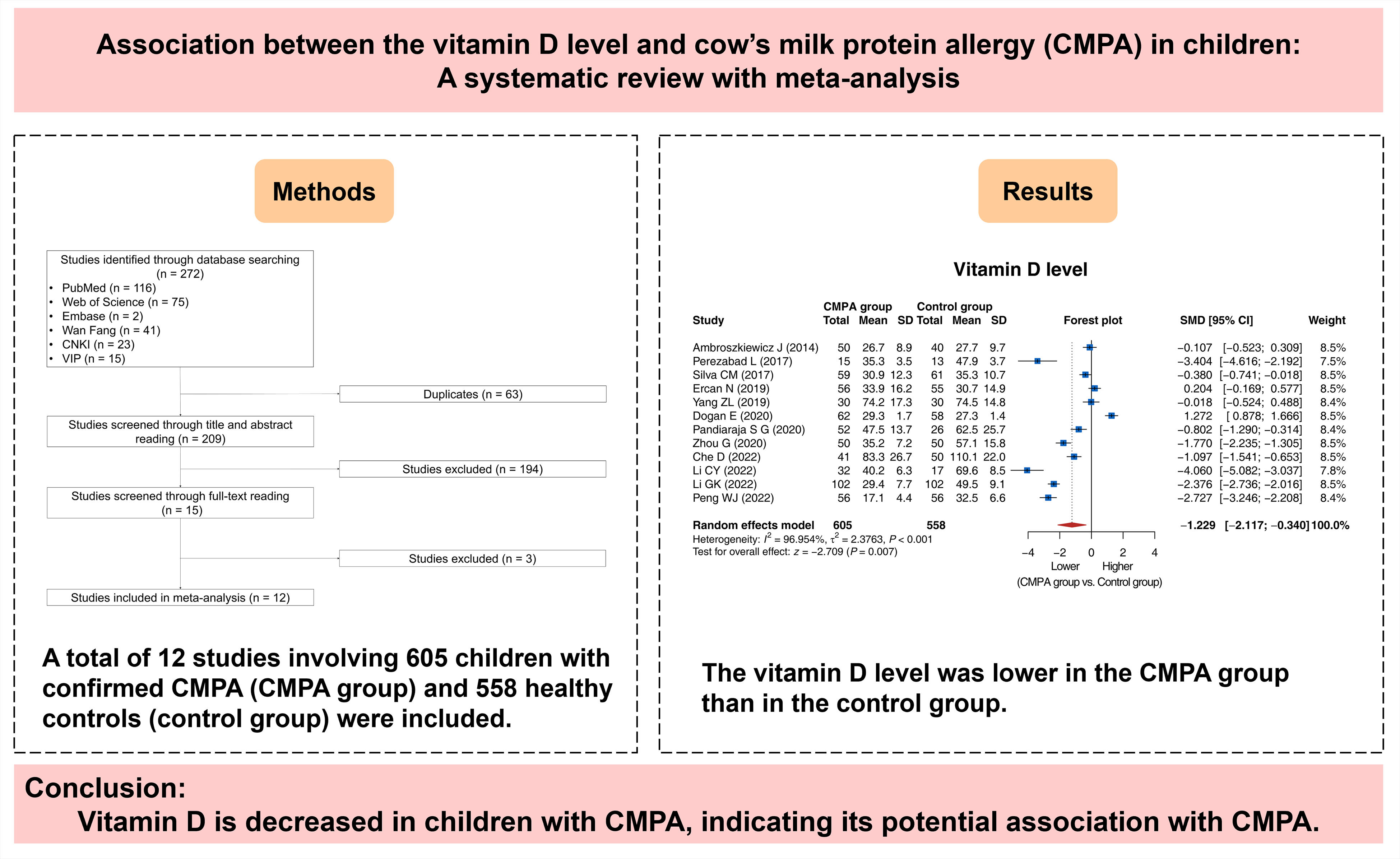

Supplement: Supplementary file 2 [file Image1.tif]
